# Supplementary material for: Genetic Analysis Reveals a Protective Effect of Sphingomyelin on Cholelithiasis
Source: Genes (Basel). 2025 Apr 29;16(5):523. doi: 10.3390/genes16050523 (PMC12110971; doi:10.3390/genes16050523)
Supplement: Supplementary file 1 [file genes-16-00523-s001.zip › Supplementary Material S1.pdf]

MR

```
library(TwoSampleMR)
library(VariantAnnotation)
library(gwasglue)
library(MRPRESSO)
a = readVcf("ieu-b-5089.vcf.gz")
b = gwasvcf_to_TwoSampleMR(vcf = a,type = "exposure")
c = subset(b, pval.exposure<5e-08)
write.csv(c,file = "exposure1.csv")
d = system.file("exposure1.csv",package="TwoSampleMR")
d = read_exposure_data(filename="exposure1.csv" ,sep = ",",snp_col = "SNP",beta_col = "beta.exposure",se_col = "se.exposure",effect_allele_col = "effect_allele.exposure",other_allele_col = "other_allele.exposure",eaf_col = "eaf.exposure",clump = TRUE)
outcome = readVcf("ukb-d-K81.vcf")
o = gwasvcf_to_TwoSampleMR(vcf = outcome,type = "outcome")
colnames(o) = c("chr.outcome","pos.outcome","other_allele.outcome","effect_allele.outcome","beta.outcome","se.outcome","pval.outcome","eaf.outcome","samplesize.outcome","ncase.outcome","SNP","ncontrol.outcome","outcome","mr_keep.outcome","pval_origin.outcome","id.outcome")
e = readVcf("",header =FALSE,sep = "\t",check.names = F,fileEncoding = "utf-8" )
f = merge(d,o,by.x = "SNP",by.y = "SNP")
write.csv(f,file = "outcome1.csv")
g = read_outcome_data(snps = f$SNP,filename='outcome1.csv',sep=",",snp_col="SNP",beta_col="beta.outcome",se_col="se.outcome",effect_allele_col="effect_allele.outcome",other_allele_col="other_allele.outcome",pval_col="pval.outcome")
h = harmonise_data(d,outcome_dat=g)
write.csv(h,file="h.csv")
h=read.csv("h.csv")
J = subset(h,pval.outcome>=5e-08)
mr_presso(BetaOutcome = "beta.outcome", BetaExposure = "beta.exposure", SdOutcome = "se.outcome", SdExposure = "se.exposure",
          OUTLIERtest = TRUE,DISTORTIONtest = TRUE, data = h, NbDistribution = 1000,
          SignifThreshold = 0.05, seed=1234)
K = subset(J,SNP != "rs1500188" & SNP != "rs3777411" & SNP != "rs77960347")
run_mr_presso(dat1,NbDistribution = 2000)
---#MR
J = read.csv("J3333.csv")
write.csv(J,file = "J3333.csv")
mr(J)
mr(h,method_list=c("mr_ivw","mr_egger_regression","mr_weighted_median"))
mr_scatter_plot(mr_results =
```

```
mr(J,method_list=c("mr_ivw","mr_egger_regression","mr_weighted_median","mr_simple_mode","mr_weighted_mode")),J)
mr_scatter_plot(mr_results =
mr(h,method_list=c("mr_ivw","mr_egger_regression","mr_weighted_median")),h)
mr_heterogeneity(J)
mr_pleiotropy_test(J)
mr_leaveoneout_plot(leaveoneout_results = mr_leaveoneout(J))
write.csv(J,file = "J444.csv")
res_single<-mr_singlesnp(J)
mr_forest_plot(res_single)
mr_funnel_plot(singlesnp_results = mr_singlesnp(J))
```

MVMR

```
library(MVMR)
library(MendelianRandomization)
library(TwoSampleMR)
id_exposure<-c("ieu-b-111","met-d-Cholines")
id_outcome<-"ebi-a-GCST90016564"
exposure_dat<-mv_extract_exposures(id_exposure)
outcome_dat<-extract_outcome_data(exposure_dat$SNP,id_outcome)
mvdat<-mv_harmonise_data(exposure_dat,outcome_dat)
SummaryStats<-cbind(mvdat[["outcome_beta"]],
                    mvdat[["exposure_beta"]][,1],
                    mvdat[["exposure_beta"]][,2],
                    mvdat[["exposure_se"]][,1],
                    mvdat[["exposure_se"]][,2],
                    mvdat[["outcome_se"]])
SummaryStats<-data.frame(SummaryStats)
MRMVInputObject_1<-mr_mvinput(
  bx=cbind(SummaryStats$X2,SummaryStats$X3),
  bxse=cbind(SummaryStats$X4,SummaryStats$X5),
  by = SummaryStats$X1,
  byse = SummaryStats$X6)
MRMVObjectivw <- mr_mvivw(MRMVInputObject_1,
                          model = "default",
                          correl = FALSE,
                          distribution = "normal",
                          alpha = 0.05)
MRMVObjectivw

MRMVObjectegger<-mr_mvegger(MRMVInputObject_1,
                          orientate = 1,
                          correl = FALSE,
```

```
                                        distribution = "normal",
                                        alpha = 0.05)
MRMVObjectegger

F.data <- format_mvmr(BXGs = SummaryStats[,c(2,3)],
                                      BYG = SummaryStats[,1],
                                      seBXGs = SummaryStats[,c(4,5)],
                                      seBYG = SummaryStats[,6])
sres <- strength_mvmr(r_input = F.data, gencov = 0)
pres <- pleiotropy_mvmr(r_input = F.data, gencov = 0)
```

Colocalization Analysis

```
library(TwoSampleMR)
library(gwasglue)
library(coloc)
library(gwasvcf)
library(dplyr)
library(gassocplot)
library(ieugwasr)
library(data.table)
library(VariantAnnotation)
library(Rfast)

reference.1000G.maf<-fread("D:/wods/reference.1000G.maf.0.005.txt.gz")

clump_data_local <- function(dat) {
   dat1<-ieugwasr::ld_clump(
      dplyr::tibble(rsid=dat$SNP, pval=dat$pval.exposure, id=dat$id.exposure),
      clump_kb = 10000,
      clump_r2 = 0.001,
      plink_bin = "D:/wods/R-4.3.0/library/plinkbinr/bin/plink_Windows.exe",
      bfile = "D:/wods/1kg.v3/EUR")
   dat2<- subset(dat,SNP %in% dat1$rsid)
   return(dat2)
}

extract_instruments_local <- function(dat, p=1e-6) {
   instruments <- subset(dat,pval < p)
   instruments <- format_data(instruments,type = "exposure")
   instruments <- clump_data_local(instruments)
   return(instruments)
}
```

```
One_sided= 50000

type1 <- "quant"
type2 <- "cc"

sample_size1 <- 114999
sample_size2 <- 361194

s1 <- 0.005
s2 <- 0.005

a = readVcf("met-d-Sphingomyelins.vcf.gz")
gwas1 = gwasvcf_to_TwoSampleMR(vcf = a,type = "exposure")
colnames(gwas1)<-c("chr","pos","other_allele","effect_allele","beta","se","pval","eaf","samplesiz
e","ncase","SNP","ncontrol","outcome","mr_keep","pval_origin","id")

b = readVcf("ukb-a-559.vcf.gz")
gwas2 = gwasvcf_to_TwoSampleMR(vcf = b,type = "outcome")
colnames(gwas2)<-c("chr","pos","other_allele","effect_allele","beta","se","pval","eaf","samplesiz
e","ncase","SNP","ncontrol","outcome","mr_keep","pval_origin","id")

gwas1<-fread("met-d-Phosphatidylc.vcf.gz")
colnames(gwas1)<-c("chr","pos","other_allele","effect_allele","SNP","neargene","pval","mlogp",
"beta","se","af","eaf","afcontrols")

gwas1<-fread("2018-07-18_SNP_AF_for_AlleleB_combined_allele_counts_and_MAF_pos_added.
txt.gz")
colnames(gwas1)<-c("chr","pos","other_allele","effect_allele","SNP","neargene","pval","mlogp",
"beta","se","af","eaf","afcontrols")

gwas2<-fread("summary_stats_finngen_R9_K11_CHOLELITH .gz")
colnames(gwas2)<-c("chr","pos","other_allele","effect_allele","SNP","neargene","pval","mlogp",
"beta","se","af","eaf","afcontrols")

gwas11 = subset(gwas1, pval<1e-06)

top = clump_data(
   gwas11,
   clump_kb = 100,
   clump_r2 = 0.001,
   clump_p1 = 1,
   clump_p2 = 1,
   pop = "EUR"
)
```

```
write.csv(top,file ="topsuise.csv" )
final_data <- data.frame(matrix(ncol = ncol(susie.res.df), nrow = 0))


for (i in 1:50) {

  chrpos <- paste0(top$chr[42], ":", top$pos[42] - One_sided*1, "-", top$pos[42] + One_sided*1)

  tab1 <- subset(gwas1, chr == top$chr[42] &
                 pos > (top$pos[42] - One_sided*1) &
                 pos < (top$pos[42] + One_sided*1)) %>%
    subset(., !duplicated(SNP))

  tab2 <- subset(gwas2, chr == top$chr[42] &
                 pos > (top$pos[42] - One_sided*1) &
                 pos < (top$pos[42] + One_sided*1))%>%
    subset(., !duplicated(SNP))

  commonsnps <- tab1$SNP[tab1$SNP %in% tab2$SNP]
  tab1 <- tab1[tab1$SNP %in% commonsnps, ] %>% dplyr::arrange(SNP)
  tab2 <- tab2[tab2$SNP %in% commonsnps, ] %>% dplyr::arrange(SNP)

  stopifnot(all(tab1$rsid == tab2$rsid))
  tab1 <- tab1 %>% format_data(type = "exposure")
  tab2 <- tab2 %>% format_data(type = "outcome")

  dat<- harmonise_data(tab1,tab2)
  colnames(dat)


tab1<-dat[,c("SNP","chr.exposure","pos.exposure","effect_allele.exposure","other_allele.exposur
e","beta.exposure",
              "se.exposure","pval.exposure","eaf.exposure")]

  colnames(tab1)<-gsub(pattern = ".exposure", replacement = "", x = colnames(tab1))


tab2<-dat[,c("SNP","chr.outcome","pos.outcome","effect_allele.outcome","other_allele.outcome
","beta.outcome",
              "se.outcome","pval.outcome","eaf.outcome")]

  colnames(tab2)<-gsub(pattern = ".outcome", replacement = "", x = colnames(tab2)
  tab1$eaf <- as.numeric(tab1$eaf)
```

```
    tab2$eaf <- as.numeric(tab2$eaf)
    tab1$eaf[which(tab1$eaf > 0.5)] <- 1 - tab1$eaf[which(tab1$eaf >
                                                                    0.5)]
    tab2$eaf[which(tab2$eaf > 0.5)] <- 1 - tab2$eaf[which(tab2$eaf >
                                                                    0.5)]
    s <- sum(is.na(tab1$eaf))
    if (s > 0) {

        tab1 <- merge(tab1, reference.1000G.maf, by = 'SNP', all.x = TRUE, suffixes = c("", ".y"))

        tab1$eaf <- ifelse(is.na(tab1$eaf), tab1$MAF, tab1$eaf)

        warning(s, " out of ", nrow(tab1), " variants have missing allele frequencies in ",
                "gwas1", ". First, match according to reference.1000G.maf. If it cannot be matched,
set it to 0.5")
        tab1$eaf[is.na(tab1$eaf)] <- 0.5
    }
    s <- sum(is.na(tab2$eaf))
    if (s > 0) {

        tab2 <- merge(tab2, reference.1000G.maf, by = 'SNP', all.x = TRUE, suffixes = c("", ".y"))

        tab2$eaf <- ifelse(is.na(tab2$eaf), tab2$MAF, tab2$eaf)

        warning(s, " out of ", nrow(tab2), " variants have missing allele frequencies in ",
                "gwas2", ". First, match according to reference.1000G.maf. If it cannot be matched,
set it to 0.5")
        tab2$eaf[is.na(tab2$eaf)] <- 0.5
    }

    tab1$sample_size1 <- sample_size1
    tab2$sample_size2 <- sample_size2


tab1 <-
    list( beta = tab1$beta, varbeta = tab1$se^2,N=tab1$sample_size1, type = type1,MAF =
tab2$eaf,
          snp = tab1$SNP,position = tab1$pos,s=s1)

tab2 <-
    list( beta = tab2$beta, varbeta = tab2$se^2,N=tab2$sample_size2, type = type2,MAF =
tab2$eaf,
           snp = tab2$SNP,position = tab2$pos,s=s2)
```

```
tab1$N = as.integer(tab1$N)
tab2$N = as.integer(tab2$N)

tab1$N <-unique(unlist(tab1$N))
tab2$N <-unique(unlist(tab2$N))


out = list(dataset1=tab1,dataset2=tab2)

out[[1]]$LD<- ld_matrix_local(out[[1]]$snp,
                              bfile = "D:/wods/1kg.v3/EUR",
                              plink_bin =
"D:/wods/R-4.3.0/library/plinkbinr/bin/plink_Windows.exe",
                              with_alleles = FALSE)

out[[2]]$LD<- ld_matrix_local(out[[2]]$snp,
                              bfile = "D:/wods/1kg.v3/EUR",
                              plink_bin =
"D:/wods/R-4.3.0/library/plinkbinr/bin/plink_Windows.exe",
                              with_alleles = FALSE)

index <- match( rownames(out[["dataset1"]][["LD"]]),    out[["dataset1"]][["snp"]] )

for (i in 1:length(names(out[["dataset1"]])) ) {

  if(length(out[["dataset1"]][[names(out[["dataset1"]])[i]]])  >1    &  !(names(out[["dataset1"]][i])
== "LD") ){

    out[["dataset1"]][[names(out[["dataset1"]])[i]]]    <-
out[["dataset1"]][[names(out[["dataset1"]])[i]]][index]

  }
}

index <- match( rownames(out[["dataset2"]][["LD"]]),    out[["dataset2"]][["snp"]] )

for (i in 1:length(names(out[["dataset2"]])) ) {

  if(length(out[["dataset2"]][[names(out[["dataset2"]])[i]]]) >1 & !(names(out[["dataset2"]][i]) ==
"LD") ){

    out[["dataset2"]][[names(out[["dataset2"]])[i]]]    <-
out[["dataset2"]][[names(out[["dataset2"]])[i]]][index]
```

```
    }
}
attach(out)

S3=runsusie(dataset1)
summary(S3)
S4=runsusie(dataset2)
summary(S4)

if(requireNamespace("susieR",quietly=TRUE)) {
    susie.res=coloc.susie(S3,S4)

}

susie.res.df<-as.data.frame(susie.res[["summary"]])
print(susie.res[["summary"]])
final_data <- rbind(final_data, susie.res.df)

  }

attach(coloc_test_data)

S3=runsusie(D3)
summary(S3)
S4=runsusie(D4)
summary(S4)

if(requireNamespace("susieR",quietly=TRUE)) {
    susie.res=coloc.susie(S3,S4)

}
```
