# Supplementary figures and images for: Genetic Analysis Reveals a Protective Effect of Sphingomyelin on Cholelithiasis
Source: Genes (Basel). 2025 Apr 29;16(5):523. doi: 10.3390/genes16050523 (PMC12110971; doi:10.3390/genes16050523)

# Genetic Analysis Reveals a Protective Effect of Sphingomyelin on Cholelithiasis

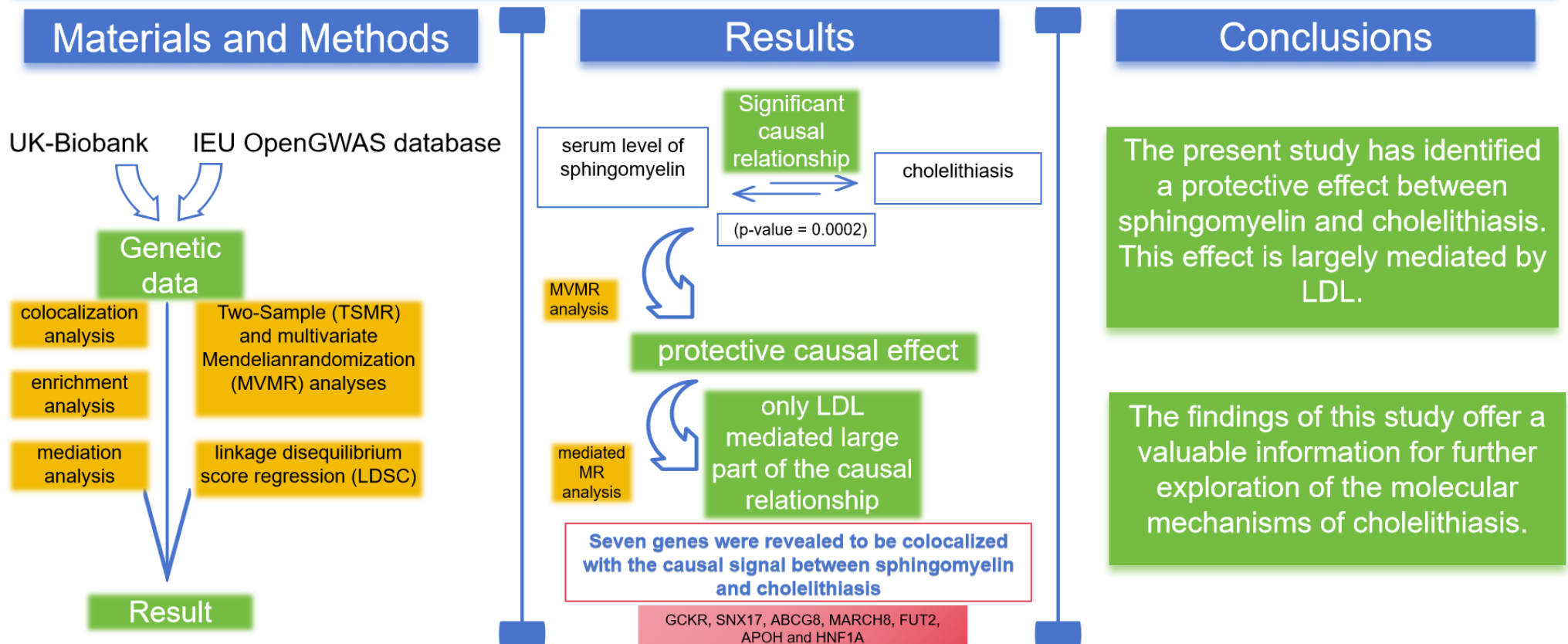

Supplement: Supplementary file 1 [file genes-16-00523-s001.zip › Supplementary Figure S1.pdf]

*A*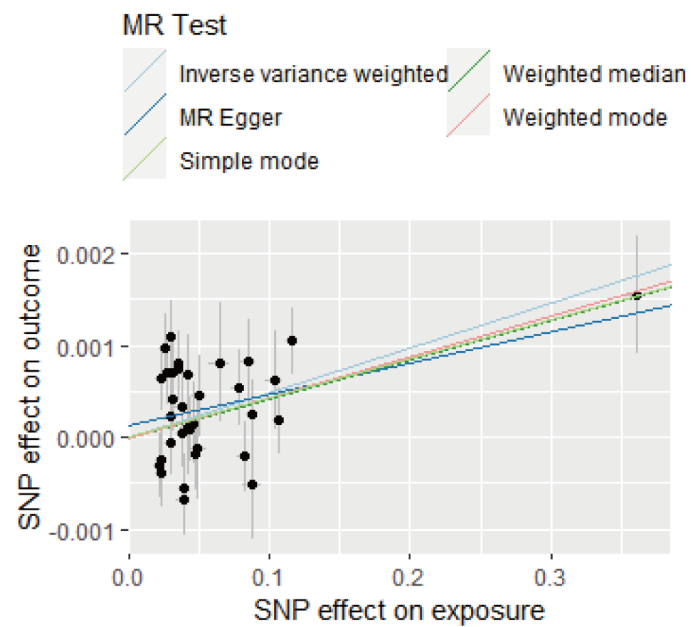*C*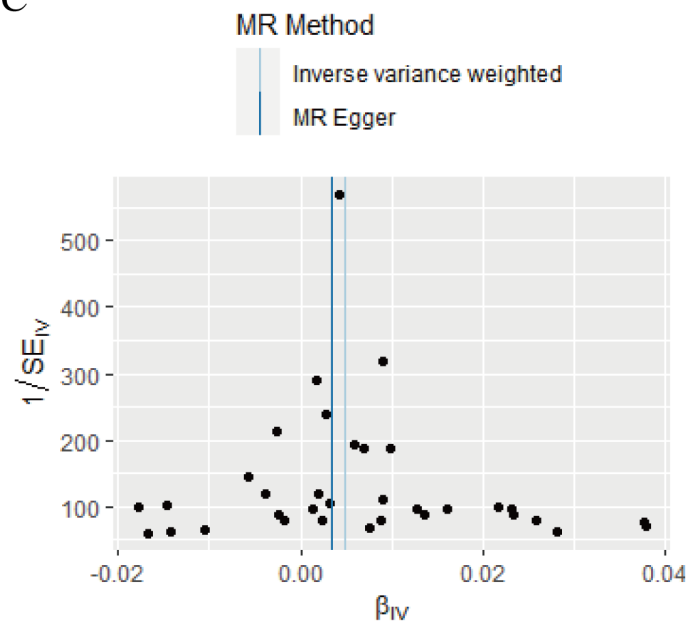*B*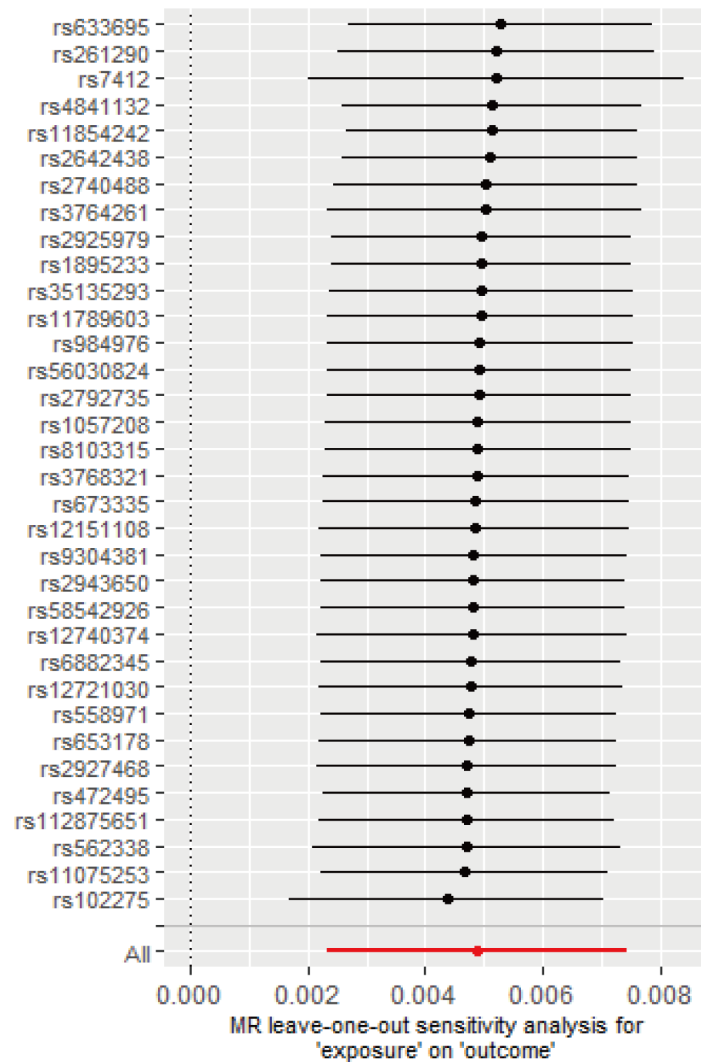

Supplement: Supplementary file 1 [file genes-16-00523-s001.zip › Supplementary Figure S2.pdf]

A

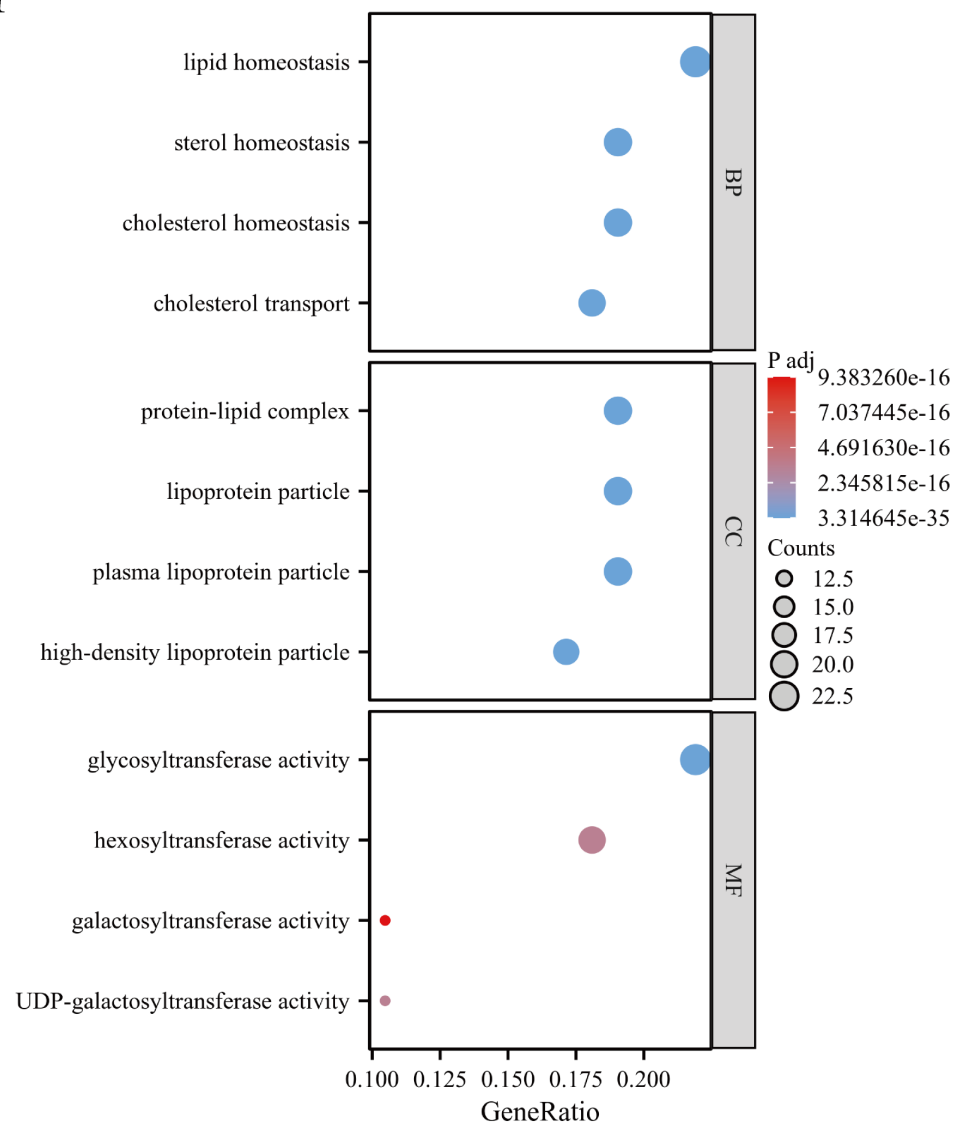

B

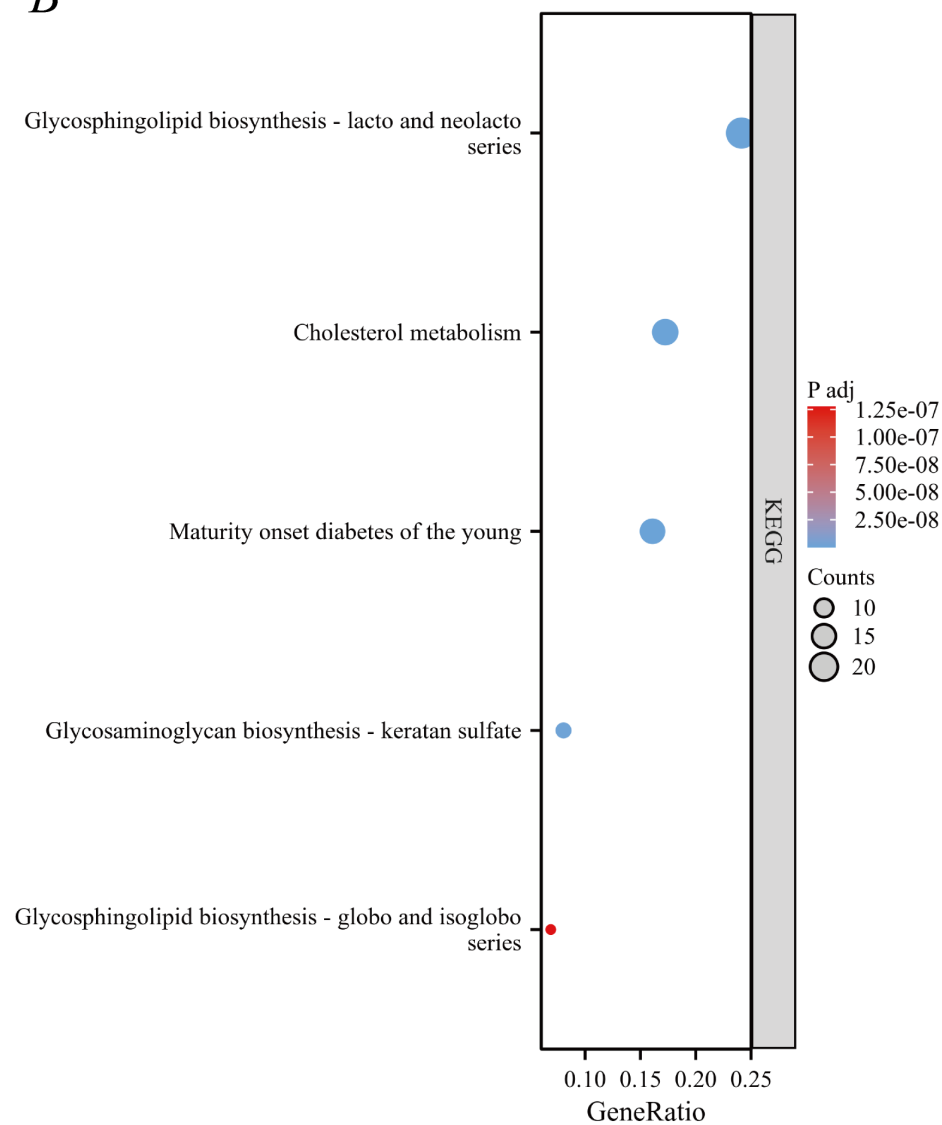

Supplement: Supplementary file 1 [file genes-16-00523-s001.zip › Supplementary Figure S3.pdf]
